# 1-(4-Chloro­phen­yl)-2-[4-hy­droxy-3-(3-meth­oxy­benzo­yl)-1,1-dioxo-2*H*-1λ^6^,2-benzothia­zin-2-yl]ethanone

**DOI:** 10.1107/S160053681201029X

**Published:** 2012-03-14

**Authors:** Hamid Latif Siddiqui, Matloob Ahmad, Salman Gul, Chaudhary Muhammad Ashraf, Masood Parvez

**Affiliations:** a Institute of Chemistry, University of the Punjab, Lahore 54590, Pakistan; b Chemistry Department, Govt. College University, Faisalabad, Pakistan; c Department of Chemistry, The University of Calgary, 2500 University Drive NW, Calgary, Alberta, Canada T2N 1N4

## Abstract

In the title mol­ecule, C_24_H_18_ClNO_6_S, the heterocyclic thia­zine ring adopts a half-chair conformation with the S and N atoms displaced by 0.406 (5) and 0.444 (5) Å, respectively, on opposite sides of the mean plane formed by the remaining ring atoms. The meth­oxy­benzoyl and the chloro­phenyl rings lie roughly parallel to each other, with a dihedral angle between the mean planes of these rings of 8.86 (10)°. The mol­ecular structure is consolidated by intra­molecular O—H⋯O and C—H⋯O inter­actions and the crystal packing is stabilized by inter­molecular O—H⋯O and C—H⋯Cl hydrogen bonds.

## Related literature

For background information on the synthesis of related compounds, see: Siddiqui *et al.* (2007 ▶). For the biological activity of benzothia­zine derivatives, see: Turck *et al.* (1995 ▶); Zia-ur-Rehman *et al.* (2006 ▶); Ahmad *et al.* (2010 ▶). For studies of benzothia­zines as precursors for azodisperse dyes for polyesters, see: Rajagopal & Seshadri (1990 ▶). For a related structure, see: Siddiqui *et al.* (2008 ▶).
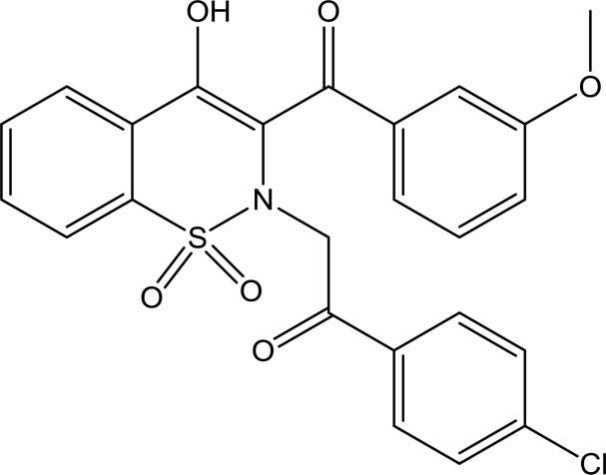


## Experimental

### 

#### Crystal data

C_24_H_18_ClNO_6_S
*M* *_r_* = 483.90
Triclinic, 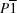

*a* = 7.2656 (2) Å
*b* = 11.4237 (4) Å
*c* = 12.8997 (5) Å
α = 97.147 (2)°
β = 96.934 (2)°
γ = 91.166 (2)°
*V* = 1053.91 (6) Å^3^
*Z* = 2
Mo *K*α radiation
μ = 0.33 mm^−1^
*T* = 123 K
0.20 × 0.12 × 0.02 mm

#### Data collection

Nonius KappaCCD diffractometer
Absorption correction: multi-scan (*SORTAV*; Blessing, 1997 ▶) *T*_min_ = 0.938, *T*_max_ = 0.994
8755 measured reflections
4729 independent reflections
3796 reflections with *I* > 2σ(*I*)
*R*_int_ = 0.041

#### Refinement

*R*[*F* ^2^ > 2σ(*F* ^2^)] = 0.054
*wR*(*F* ^2^) = 0.111
*S* = 1.09
4729 reflections
300 parameters
H-atom parameters constrained
Δρ_max_ = 0.40 e Å^−3^
Δρ_min_ = −0.46 e Å^−3^

### 

Data collection: *COLLECT* (Hooft, 1998 ▶); cell refinement: *DENZO* (Otwinowski & Minor, 1997 ▶); data reduction: *SCALEPACK* (Otwinowski & Minor, 1997 ▶); program(s) used to solve structure: *SHELXS97* (Sheldrick, 2008 ▶); program(s) used to refine structure: *SHELXL97* (Sheldrick, 2008 ▶); molecular graphics: *ORTEP-3 for Windows* (Farrugia, 1997 ▶); software used to prepare material for publication: *SHELXL97*.

## Supplementary Material

Crystal structure: contains datablock(s) global, I. DOI: 10.1107/S160053681201029X/pk2395sup1.cif

Structure factors: contains datablock(s) I. DOI: 10.1107/S160053681201029X/pk2395Isup2.hkl

Supplementary material file. DOI: 10.1107/S160053681201029X/pk2395Isup3.cml

Additional supplementary materials:  crystallographic information; 3D view; checkCIF report

## Figures and Tables

**Table 1:** Hydrogen-bond geometry (Å, °)

*D*—H⋯*A*	*D*—H	H⋯*A*	*D*⋯*A*	*D*—H⋯*A*
O3—H3*O*⋯O4^i^	0.84	2.32	2.926 (3)	129
C3—H3⋯Cl1^ii^	0.95	2.78	3.711 (3)	167
O3—H3*O*⋯O4	0.84	1.81	2.546 (3)	145
C24—H24⋯O2	0.95	2.60	3.534 (3)	169

Symmetry codes: (i) 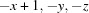; (ii) 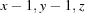.

**Fig. 1.**
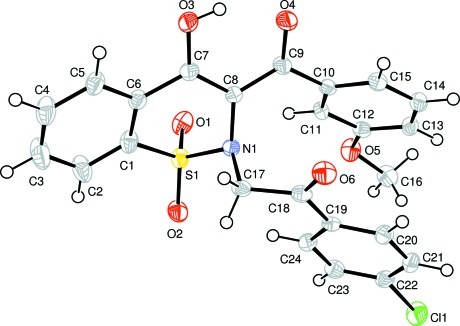
The molecular structure of the title compound with displacement ellipsoids drawn at the 50% probability level. H atoms are presented as small spheres of arbitrary radius.

**Fig. 2.**
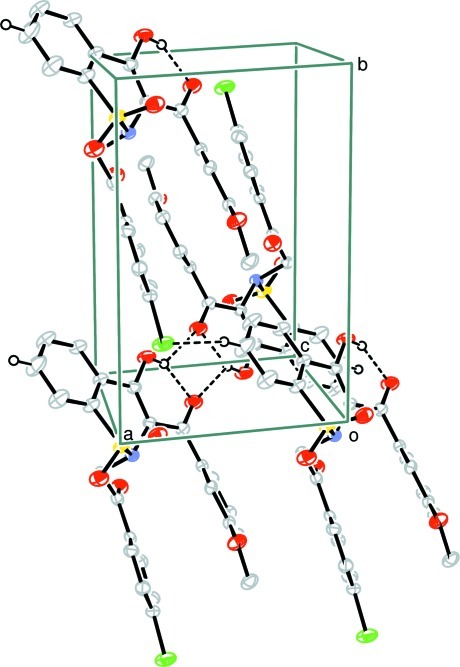
A view of the C—-H···O and C—H···Cl hydrogen bonds (dotted lines) in the crystal structure of the title compound. H atoms non-participating in hydrogen-bonding have been omitted for clarity.

## supplementary crystallographic information

### Comment

Derivatives of benzothiazine have been studied for a broad range of biological activities. They are found to possess analgesic (Turck *et al*., 1995), antimicrobial (Zia-ur-Rehman *et al.*, 2006) and antioxidant activities (Ahmad *et al.*, 2010), *etc*. A few benzothiazines have also been studied as precursors for azodisperse dyes for polyesters (Rajagopal & Seshadri 1990). In continuation of our research on the synthesis of biologically active benzothiazine derivatives (Siddiqui *et al.*, 2007; Ahmad *et al.*, 2010), we herein report the synthesis and crystal structure of the title compound.

The bond distances and angles in the title compound (Fig. 1) agree very well with the corresponding bond distances and angles reported in closely related compounds (Siddiqui *et al.*, 2008). The heterocyclic thiazine ring adopts a half chair conformation with the S1 and N1 atoms displaced by 0.406 (5) and 0.444 (5) Å, respectively, on opposite sides of the mean plane formed by the remaining ring atoms. The methoxybenzoyl and the chlorophenyl rings lie roughly parallel to each other with a dihedral angle between the mean planes of these rings of 8.86 (10)°; the distance between the centroids of these rings is 3.828 (14) Å. The molecular structure of the title compound is consolidated by intramolecular interactions O3–H3O···O4 and C24—H24···O2 and the crystal packing is stabilized by intermolecular O3—H3O···O4 and C3—H3···Cl1 hydrogen bonds (Fig. 2 and Table 1).

### Experimental

A mixture of (4-hydroxy-1,1-dioxido-2*H*-1,2-benzothiazin-3-yl)(3-methoxyphenyl) methanone (5.0 g, 0.015 mol), K_2_CO_3_ (2.07 g, 0.015 mol) and 4-chlorophenacyl bromide (3.50 g, 0.015 mol) in acetonitrile (30 ml) was refluxed for 3 h. The contents of the flask were poured on ice cold HCl (5%, 30 ml). The precipitate of the title compound thus formed was collected and washed with ethanol. Crystals suitable for X-ray crystallographic analysis were grown from a solution in methanol.

### Refinement

All H atoms were positioned geometrically and refined using a riding model, with O—H = 0.84 Å and C—H = 0.95, 0.98 and 0.99 Å, for aryl, methyl and methylene H-atoms, respectively. The *U*_iso_(H) were allowed at 1.5*U*_eq_(O) or 1.2*U*_eq_(C).

### Crystal data

**Table:**

C_24_H_18_ClNO_6_S	*Z* = 2
*M**_r_* = 483.90	*F*(000) = 500
Triclinic, *P*1	*D*_x_ = 1.525 Mg m^−^^3^
Hall symbol: -P 1	Mo *K*α radiation, λ = 0.71073 Å
*a* = 7.2656 (2) Å	Cell parameters from 4426 reflections
*b* = 11.4237 (4) Å	θ = 1.0–27.5°
*c* = 12.8997 (5) Å	µ = 0.33 mm^−^^1^
α = 97.147 (2)°	*T* = 123 K
β = 96.934 (2)°	Plate, colorless
γ = 91.166 (2)°	0.20 × 0.12 × 0.02 mm
*V* = 1053.91 (6) Å^3^	

### Data collection

**Table:**

Nonius KappaCCD diffractometer	4729 independent reflections
Radiation source: fine-focus sealed tube	3796 reflections with *I* > 2σ(*I*)
Graphite monochromator	*R*_int_ = 0.041
ω and φ scans	θ_max_ = 27.5°, θ_min_ = 3.2°
Absorption correction: multi-scan (*SORTAV*; Blessing, 1997)	*h* = −9→9
*T*_min_ = 0.938, *T*_max_ = 0.994	*k* = −14→14
8755 measured reflections	*l* = −16→16

### Refinement

**Table:**

Refinement on *F*^2^	Primary atom site location: structure-invariant direct methods
Least-squares matrix: full	Secondary atom site location: difference Fourier map
*R*[*F*^2^ > 2σ(*F*^2^)] = 0.054	Hydrogen site location: inferred from neighbouring sites
*wR*(*F*^2^) = 0.111	H-atom parameters constrained
*S* = 1.09	*w* = 1/[σ^2^(*F*_o_^2^) + (0.0049*P*)^2^ + 2.1351*P*] where *P* = (*F*_o_^2^ + 2*F*_c_^2^)/3
4729 reflections	(Δ/σ)_max_ < 0.001
300 parameters	Δρ_max_ = 0.40 e Å^−^^3^
0 restraints	Δρ_min_ = −0.46 e Å^−^^3^

### Special details

**Table:**

Geometry. All e.s.d.'s (except the e.s.d. in the dihedral angle between two l.s. planes) are estimated using the full covariance matrix. The cell e.s.d.'s are taken into account individually in the estimation of e.s.d.'s in distances, angles and torsion angles; correlations between e.s.d.'s in cell parameters are only used when they are defined by crystal symmetry. An approximate (isotropic) treatment of cell e.s.d.'s is used for estimating e.s.d.'s involving l.s. planes.
Refinement. Refinement of *F*^2^ against ALL reflections. The weighted *R*-factor *wR* and goodness of fit *S* are based on *F*^2^, conventional *R*-factors *R* are based on *F*, with *F* set to zero for negative *F*^2^. The threshold expression of *F*^2^ > σ(*F*^2^) is used only for calculating *R*-factors(gt) *etc*. and is not relevant to the choice of reflections for refinement. *R*-factors based on *F*^2^ are statistically about twice as large as those based on *F*, and *R*- factors based on ALL data will be even larger.

### Fractional atomic coordinates and isotropic or equivalent isotropic displacement parameters (Å^2^)

**Table:**

	*x*	*y*	*z*	*U*_iso_*/*U*_eq_	
Cl1	0.27652 (11)	0.84583 (6)	0.39721 (6)	0.02956 (18)	
S1	0.08312 (9)	0.16251 (6)	0.34160 (5)	0.01952 (15)	
O1	0.2480 (3)	0.12646 (17)	0.39870 (15)	0.0259 (4)	
O2	−0.0328 (3)	0.24442 (17)	0.39351 (15)	0.0249 (4)	
O3	0.2434 (3)	−0.05681 (16)	0.07934 (16)	0.0243 (4)	
H3O	0.3375	−0.0270	0.0593	0.036*	
O4	0.4778 (3)	0.10707 (16)	0.06442 (16)	0.0253 (4)	
O5	0.6259 (3)	0.48812 (16)	0.41015 (15)	0.0240 (4)	
O6	0.1052 (3)	0.36792 (17)	0.03952 (14)	0.0227 (4)	
N1	0.1451 (3)	0.21848 (18)	0.23821 (17)	0.0164 (4)	
C1	−0.0525 (4)	0.0389 (2)	0.2786 (2)	0.0206 (6)	
C2	−0.2090 (4)	0.0013 (3)	0.3183 (2)	0.0313 (7)	
H2	−0.2473	0.0428	0.3801	0.038*	
C3	−0.3088 (5)	−0.0976 (3)	0.2662 (3)	0.0375 (8)	
H3	−0.4161	−0.1248	0.2928	0.045*	
C4	−0.2535 (4)	−0.1572 (3)	0.1758 (2)	0.0303 (7)	
H4	−0.3233	−0.2251	0.1409	0.036*	
C5	−0.0983 (4)	−0.1195 (2)	0.1354 (2)	0.0237 (6)	
H5	−0.0618	−0.1611	0.0732	0.028*	
C6	0.0048 (4)	−0.0198 (2)	0.1867 (2)	0.0192 (5)	
C7	0.1702 (4)	0.0231 (2)	0.1447 (2)	0.0190 (5)	
C8	0.2433 (3)	0.1361 (2)	0.1725 (2)	0.0163 (5)	
C9	0.4103 (4)	0.1743 (2)	0.1327 (2)	0.0184 (5)	
C10	0.4997 (3)	0.2931 (2)	0.1685 (2)	0.0168 (5)	
C11	0.5250 (3)	0.3394 (2)	0.2740 (2)	0.0178 (5)	
H11	0.4856	0.2948	0.3252	0.021*	
C12	0.6081 (3)	0.4512 (2)	0.3051 (2)	0.0179 (5)	
C13	0.6657 (3)	0.5170 (2)	0.2303 (2)	0.0188 (5)	
H13	0.7200	0.5939	0.2510	0.023*	
C14	0.6429 (4)	0.4690 (2)	0.1249 (2)	0.0206 (6)	
H14	0.6834	0.5133	0.0737	0.025*	
C15	0.5621 (3)	0.3577 (2)	0.0934 (2)	0.0193 (5)	
H15	0.5490	0.3252	0.0213	0.023*	
C16	0.6796 (4)	0.6090 (2)	0.4448 (2)	0.0257 (6)	
H16A	0.6735	0.6261	0.5206	0.031*	
H16B	0.5954	0.6599	0.4071	0.031*	
H16C	0.8067	0.6239	0.4303	0.031*	
C17	−0.0038 (3)	0.2799 (2)	0.1785 (2)	0.0182 (5)	
H17A	−0.0939	0.3108	0.2259	0.022*	
H17B	−0.0708	0.2237	0.1210	0.022*	
C18	0.0834 (3)	0.3813 (2)	0.1327 (2)	0.0188 (5)	
C19	0.1369 (3)	0.4937 (2)	0.2017 (2)	0.0174 (5)	
C20	0.1938 (3)	0.5903 (2)	0.1556 (2)	0.0194 (5)	
H20	0.2023	0.5816	0.0821	0.023*	
C21	0.2381 (4)	0.6983 (2)	0.2152 (2)	0.0216 (6)	
H21	0.2760	0.7640	0.1834	0.026*	
C22	0.2262 (4)	0.7091 (2)	0.3228 (2)	0.0208 (6)	
C23	0.1736 (4)	0.6146 (2)	0.3712 (2)	0.0204 (5)	
H23	0.1694	0.6232	0.4451	0.025*	
C24	0.1272 (4)	0.5073 (2)	0.3102 (2)	0.0189 (5)	
H24	0.0884	0.4421	0.3423	0.023*	

### Atomic displacement parameters (Å^2^)

**Table:**

	*U*^11^	*U*^22^	*U*^33^	*U*^12^	*U*^13^	*U*^23^
Cl1	0.0383 (4)	0.0188 (3)	0.0307 (4)	−0.0049 (3)	0.0109 (3)	−0.0061 (3)
S1	0.0242 (3)	0.0169 (3)	0.0169 (3)	−0.0042 (2)	0.0053 (3)	−0.0019 (2)
O1	0.0325 (11)	0.0223 (10)	0.0219 (10)	−0.0043 (8)	0.0000 (8)	0.0033 (8)
O2	0.0308 (11)	0.0224 (10)	0.0216 (10)	−0.0046 (8)	0.0120 (8)	−0.0047 (8)
O3	0.0277 (11)	0.0173 (10)	0.0282 (11)	−0.0027 (8)	0.0123 (9)	−0.0042 (8)
O4	0.0288 (11)	0.0194 (10)	0.0285 (11)	−0.0001 (8)	0.0143 (9)	−0.0048 (8)
O5	0.0311 (11)	0.0205 (10)	0.0191 (10)	−0.0030 (8)	0.0034 (8)	−0.0018 (8)
O6	0.0262 (10)	0.0241 (10)	0.0175 (10)	0.0008 (8)	0.0038 (8)	−0.0004 (8)
N1	0.0167 (10)	0.0129 (10)	0.0195 (11)	−0.0018 (8)	0.0048 (8)	−0.0007 (8)
C1	0.0244 (14)	0.0163 (13)	0.0200 (14)	−0.0057 (10)	0.0042 (11)	−0.0018 (10)
C2	0.0358 (17)	0.0320 (17)	0.0262 (16)	−0.0103 (13)	0.0137 (13)	−0.0040 (13)
C3	0.0399 (19)	0.0396 (19)	0.0328 (18)	−0.0191 (15)	0.0130 (14)	−0.0014 (14)
C4	0.0330 (16)	0.0261 (16)	0.0296 (16)	−0.0137 (12)	0.0014 (13)	−0.0001 (13)
C5	0.0286 (15)	0.0198 (14)	0.0218 (14)	−0.0042 (11)	0.0028 (11)	−0.0001 (11)
C6	0.0218 (13)	0.0152 (13)	0.0204 (13)	−0.0021 (10)	0.0035 (10)	0.0014 (10)
C7	0.0228 (13)	0.0167 (13)	0.0168 (13)	0.0018 (10)	0.0015 (10)	−0.0004 (10)
C8	0.0170 (12)	0.0146 (12)	0.0178 (13)	0.0008 (9)	0.0054 (10)	0.0008 (10)
C9	0.0200 (13)	0.0164 (13)	0.0184 (13)	0.0015 (10)	0.0024 (10)	0.0005 (10)
C10	0.0144 (12)	0.0159 (13)	0.0205 (13)	0.0022 (9)	0.0040 (10)	0.0011 (10)
C11	0.0168 (12)	0.0178 (13)	0.0194 (13)	0.0019 (10)	0.0049 (10)	0.0023 (10)
C12	0.0164 (12)	0.0196 (13)	0.0169 (13)	0.0028 (10)	0.0019 (10)	−0.0018 (10)
C13	0.0170 (12)	0.0179 (13)	0.0211 (14)	0.0003 (10)	0.0012 (10)	0.0018 (10)
C14	0.0190 (13)	0.0236 (14)	0.0204 (14)	0.0011 (10)	0.0040 (11)	0.0062 (11)
C15	0.0174 (12)	0.0226 (14)	0.0171 (13)	−0.0003 (10)	0.0029 (10)	−0.0010 (10)
C16	0.0257 (14)	0.0248 (15)	0.0239 (15)	−0.0054 (11)	0.0046 (11)	−0.0089 (12)
C17	0.0156 (12)	0.0184 (13)	0.0195 (13)	0.0001 (10)	0.0010 (10)	−0.0007 (10)
C18	0.0152 (12)	0.0197 (13)	0.0209 (14)	0.0032 (10)	0.0010 (10)	0.0015 (10)
C19	0.0158 (12)	0.0163 (13)	0.0194 (13)	0.0034 (9)	0.0012 (10)	0.0006 (10)
C20	0.0185 (12)	0.0241 (14)	0.0161 (13)	0.0032 (10)	0.0032 (10)	0.0033 (10)
C21	0.0210 (13)	0.0191 (13)	0.0258 (15)	0.0008 (10)	0.0061 (11)	0.0045 (11)
C22	0.0180 (13)	0.0177 (13)	0.0255 (14)	0.0011 (10)	0.0037 (11)	−0.0032 (11)
C23	0.0204 (13)	0.0215 (14)	0.0187 (13)	0.0010 (10)	0.0028 (10)	−0.0001 (11)
C24	0.0203 (13)	0.0183 (13)	0.0191 (13)	0.0015 (10)	0.0049 (10)	0.0033 (10)

### Geometric parameters (Å, º)

**Table:**

Cl1—C22	1.736 (3)	C10—C11	1.387 (4)
S1—O1	1.425 (2)	C10—C15	1.398 (4)
S1—O2	1.4302 (19)	C11—C12	1.392 (4)
S1—N1	1.654 (2)	C11—H11	0.9500
S1—C1	1.758 (3)	C12—C13	1.391 (4)
O3—C7	1.328 (3)	C13—C14	1.390 (4)
O3—H3O	0.8400	C13—H13	0.9500
O4—C9	1.247 (3)	C14—C15	1.382 (4)
O5—C12	1.358 (3)	C14—H14	0.9500
O5—C16	1.429 (3)	C15—H15	0.9500
O6—C18	1.222 (3)	C16—H16A	0.9800
N1—C8	1.445 (3)	C16—H16B	0.9800
N1—C17	1.490 (3)	C16—H16C	0.9800
C1—C2	1.383 (4)	C17—C18	1.524 (4)
C1—C6	1.400 (4)	C17—H17A	0.9900
C2—C3	1.384 (4)	C17—H17B	0.9900
C2—H2	0.9500	C18—C19	1.484 (4)
C3—C4	1.383 (4)	C19—C20	1.395 (4)
C3—H3	0.9500	C19—C24	1.399 (4)
C4—C5	1.380 (4)	C20—C21	1.380 (4)
C4—H4	0.9500	C20—H20	0.9500
C5—C6	1.398 (4)	C21—C22	1.392 (4)
C5—H5	0.9500	C21—H21	0.9500
C6—C7	1.476 (4)	C22—C23	1.382 (4)
C7—C8	1.374 (4)	C23—C24	1.384 (4)
C8—C9	1.453 (3)	C23—H23	0.9500
C9—C10	1.488 (4)	C24—H24	0.9500
			
O1—S1—O2	119.52 (12)	O5—C12—C11	115.4 (2)
O1—S1—N1	107.09 (11)	C13—C12—C11	120.1 (2)
O2—S1—N1	108.26 (11)	C14—C13—C12	119.4 (2)
O1—S1—C1	110.53 (13)	C14—C13—H13	120.3
O2—S1—C1	109.33 (12)	C12—C13—H13	120.3
N1—S1—C1	100.32 (12)	C15—C14—C13	120.9 (2)
C7—O3—H3O	109.5	C15—C14—H14	119.5
C12—O5—C16	117.7 (2)	C13—C14—H14	119.5
C8—N1—C17	113.8 (2)	C14—C15—C10	119.5 (2)
C8—N1—S1	112.71 (17)	C14—C15—H15	120.2
C17—N1—S1	115.04 (16)	C10—C15—H15	120.2
C2—C1—C6	121.5 (2)	O5—C16—H16A	109.5
C2—C1—S1	121.3 (2)	O5—C16—H16B	109.5
C6—C1—S1	117.13 (19)	H16A—C16—H16B	109.5
C1—C2—C3	118.8 (3)	O5—C16—H16C	109.5
C1—C2—H2	120.6	H16A—C16—H16C	109.5
C3—C2—H2	120.6	H16B—C16—H16C	109.5
C4—C3—C2	120.5 (3)	N1—C17—C18	109.1 (2)
C4—C3—H3	119.8	N1—C17—H17A	109.9
C2—C3—H3	119.8	C18—C17—H17A	109.9
C5—C4—C3	120.9 (3)	N1—C17—H17B	109.9
C5—C4—H4	119.5	C18—C17—H17B	109.9
C3—C4—H4	119.5	H17A—C17—H17B	108.3
C4—C5—C6	119.6 (3)	O6—C18—C19	121.9 (2)
C4—C5—H5	120.2	O6—C18—C17	118.8 (2)
C6—C5—H5	120.2	C19—C18—C17	119.3 (2)
C5—C6—C1	118.7 (2)	C20—C19—C24	118.9 (2)
C5—C6—C7	120.7 (2)	C20—C19—C18	118.4 (2)
C1—C6—C7	120.6 (2)	C24—C19—C18	122.6 (2)
O3—C7—C8	123.0 (2)	C21—C20—C19	121.0 (2)
O3—C7—C6	114.5 (2)	C21—C20—H20	119.5
C8—C7—C6	122.5 (2)	C19—C20—H20	119.5
C7—C8—N1	118.4 (2)	C20—C21—C22	118.7 (2)
C7—C8—C9	121.1 (2)	C20—C21—H21	120.7
N1—C8—C9	120.4 (2)	C22—C21—H21	120.7
O4—C9—C8	119.2 (2)	C23—C22—C21	121.8 (2)
O4—C9—C10	119.0 (2)	C23—C22—Cl1	119.5 (2)
C8—C9—C10	121.7 (2)	C21—C22—Cl1	118.6 (2)
C11—C10—C15	119.9 (2)	C22—C23—C24	118.8 (2)
C11—C10—C9	121.5 (2)	C22—C23—H23	120.6
C15—C10—C9	118.6 (2)	C24—C23—H23	120.6
C10—C11—C12	120.2 (2)	C23—C24—C19	120.8 (2)
C10—C11—H11	119.9	C23—C24—H24	119.6
C12—C11—H11	119.9	C19—C24—H24	119.6
O5—C12—C13	124.6 (2)		

### Hydrogen-bond geometry (Å, º)

**Table:**

*D*—H···*A*	*D*—H	H···*A*	*D*···*A*	*D*—H···*A*
O3—H3*O*···O4^i^	0.84	2.32	2.926 (3)	129
C3—H3···Cl1^ii^	0.95	2.78	3.711 (3)	167
O3—H3*O*···O4	0.84	1.81	2.546 (3)	145
C17—H17*A*···O2	0.99	2.37	2.885 (3)	111
C24—H24···O2	0.95	2.60	3.534 (3)	169

Symmetry codes: (i) −*x*+1, −*y*, −*z*; (ii) *x*−1, *y*−1, *z*.
